# Social Isolation and Accelerated Tooth Loss Among Chinese Older Adults

**DOI:** 10.1093/geroni/igab046.112

**Published:** 2021-12-17

**Authors:** Xiang Qi, Yaolin Pei, Katherine Wang, Shuyu Han, Bei Wu

**Affiliations:** 1 Rory Meyers College of nursing, New York, New York, United States; 2 New York University, New York, New York, United States; 3 Duke University, Durham, North Carolina, United States; 4 Fudan University, Shanghai, Shanghai, China (People's Republic)

## Abstract

Social isolation and loneliness in older adults are major global public health concerns. Tooth loss is also a common problem in this population. This study examined the effects of social isolation and loneliness on the number of remaining teeth and the rate of tooth loss among Chinese older adults. We included 4,268 older adults age 65+ from three waves of the Chinese Longitudinal Healthy Longevity Survey (2011/2012, 2014, 2018). Linear mixed-effect models showed higher levels of social isolation were associated with fewer remaining teeth (β = -1.59, P < 0.05) and accelerated tooth loss (β=-0.10, P<0.05) controlled for socio-demographic, lifestyle, oral hygiene behavior, and health status. Loneliness was neither associated with the number of remaining teeth (β=0.64, P>0.05) nor with the rate of tooth loss (β=-0.09, P>0.05) before and after controlling for covariates. These findings expand our knowledge regarding the correlation between social connection and tooth loss in non-Western populations.

